# Illumina-MiSeq analysis of fungi in acid lime roots reveals dominance of *Fusarium* and variation in fungal taxa

**DOI:** 10.1038/s41598-018-35404-5

**Published:** 2018-11-26

**Authors:** Abdullah M. Al-Sadi, Elham A. Kazerooni

**Affiliations:** Department of Crop Sciences, College of Agricultural and Marine Sciences, Sultan Qaboos University, Oman, PO Box 34, Alkhoud, 123 Oman

## Abstract

A study was conducted to analyze fungal diversity in the roots of acid lime (*Citrus aurantifolia*) collected from Oman, a semi-arid country located in the South Eastern part of the Arabian Peninsula. MiSeq analysis showed the *Ascomycota* and *Sordariomycetes* were the most abundant phylum and class in acid lime roots, respectively. *Glomeromycota, Basidiomycota* and *Microsporidia* were the other fungal phyla, while *Glomeromycetes* and some other classes belonging to *Ascomycota* and *Basidiomycota* were detected at lower frequencies. The genus *Fusarium* was the most abundant in all samples, making up 46 to 95% of the total reads. Some fungal genera of Arbuscular mycorrhizae and nematophagous fungi were detected in some of the acid lime roots. Analysis of the level of fungal diversity showed that no significant differences exist among groups of root samples (from different locations) in their Chao richness and Shannon diversity levels (P < 0.05). Principle component analysis of fungal communities significantly separated samples according to their locations. This is the first study to evaluate fungal diversity in acid lime roots using high throughput sequencing analysis. The study reveals the presence of various fungal taxa in the roots, dominated by *Fusarium* species and including some mycorrhizae and nematophagous fungi.

## Introduction

Plants depend on soil microorganisms for recycling nutrients, while soil microbes rely on plants for their energy requirements. Plants release their photosynthesis products and root exudates into soil and by this way they enhance microbial growth rates and assembly^1^. For example, arbuscular mycorrhizal fungi produce a substance called glomalin that improves soil structure, stabilizes soil aggregates and prepares more favorable structure for root growth^2^. Different plant genotypes and cultivars can be favorable for various saprophytic and pathogenic microorganisms^2,3^.

Fungi have a wider geographical distribution compared to plants and other organisms^4^. Among soil microbial community, fungi take place after bacteria as the second most abundant group of soil microbiota. Some fungi are responsible for decomposing plant residues and organic materials by releasing enzymes which breakdown all these material to absorbable form for fungi. Improving soil structure and aeration is another role of the soil fungi. Some of them may form mutualistic relationship with plant and help the plant to uptake more nutrients^5^.

The term endophytic fungi refers to systemic symbiotic fungi that occupy living plant tissues without causing any pathogenic effect. Depending on host species and fungi, endophytes play different ecological roles in different fields such as protecting their host against herbivores, pathogenic organisms and drought stress. They can also increase nitrogen uptake and stimulate root growth^6–10^. Endophytes are capable of enhancing plant growth and resistance by different mechanisms such as nitrogen fixation, increasing phosphorus uptake and production of plant hormones and siderophores.

Acid lime (*Citrus aurantifolia*) is an important crop in different parts of the world. Total production of acid limes reached 17 million tons in 2016^11^. In Oman, acid lime is among the top four fruit crops in terms of production. The cultivation system in most parts of the country is traditional, where different crops are grown in the same farm, with date palms, acid limes and mangoes being common in most farms^12^.

Studies of the fungal species associated with roots of different crops is an important step towards understanding the roles played by fungi in the roots. Several studies addressed fungal root endophytes in various crops^9,13–16^. However, studies on fungi associated with citrus roots are very limited^17-19^. In addition, studies on fungi associated with roots of acid limes are even more limited to only few studies on mycorrhizae and pathogenic fungi^19–21^. Lack of knowledge in this area makes it difficult to understand the roles played by fungi in the root system of acid limes.

Detection of fungi in plant roots has long relied on culture-based techniques^13,21–24^. However, these techniques have been limited by their inability to recover obligate fungi, creating a gap in knowledge about the various fungal species associated with the root system. Illumina MiSeq technique has provided a powerful tool for the analysis of fungal communities in different substrates and plant tissues. It is able to detect culturable and non-culturable fungal communities^25–28^.

The aim of this study was to study fungal communities associated with roots of acid lime in five locations in Oman using MiSeq analysis. The study will help come up with a list of potential fungal taxa present in the roots and how they vary over locations.

## Results

### Diversity estimates

The observed OTUs ranged from 14 to 80. Chao1 richness estimates of the 17 samples obtained from five locations in Oman showed that values range from 14 to 85 for the 17 individual samples (Supplementary Fig. S1). The mean values from each of the five locations ranged from 28 to 61, with no significant differences between the locations in richness estimates (P ≤ 0.5; Supplementary Fig. S1). Shannon estimates ranged from 0.1 to 1.6 for individual samples and 0.3 to 1.2 among the five main locations, with no significant differences between locations in Shannon estimates (P ≤ 0.5; Supplementary Fig. S2). The lack of significant differences was mainly due to the high variation among samples obtained from the same location.

### Fungal Classes and Genera

**Figure 1:**
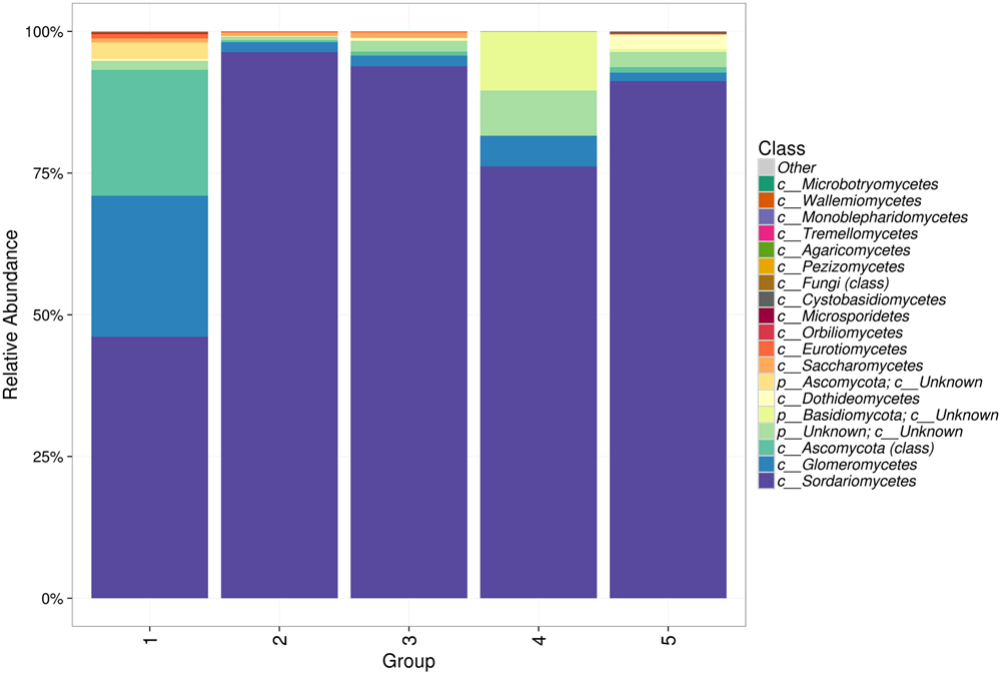
Class-level relative abundance of fungal communities in the five groups of acid lime roots.

Acid lime roots were mainly dominated by fungal communities belonging to *Ascomycota* phylum. *Glomeromycota, Basidiomycota* and *Microsporidia* were also present but at lower frequencies. The most dominant class across all groups was *Sordarioymcetes*, making up over 90% of three groups (2, 3, and 5) and 76% of group 4 and 46% of group 1. *Glomeromycetes* was the second most common followed by unknown classes from *Ascomycota* and *Basidiomycota*. The remaining classes were present at low frequencies (Fig. 1). Comparison across groups from different locations showed that most groups of root samples share the same classes.

**Figure 2:**
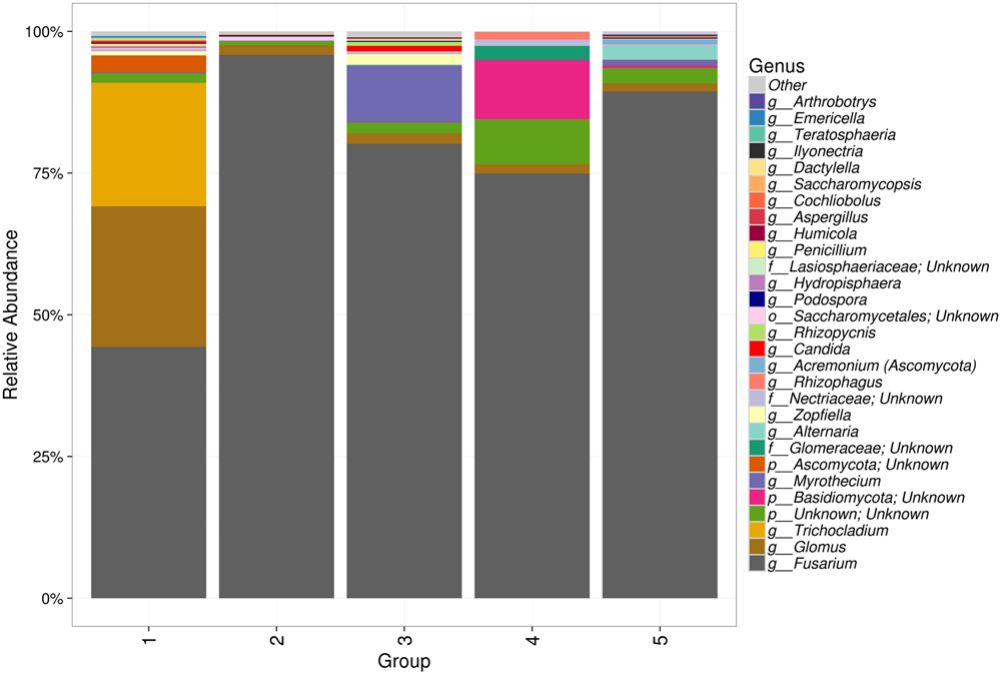
Generic-level relative abundance of fungal communities in the five groups of acid lime roots.

The genus *Fusarium* was the most abundant in all samples, present at frequencies of 46 to 95% in the samples. *Glomus* and *Trichocladium* were the two other abundant genera in group 1. *Arthrobotrys* and an unknown genus belonging to *Basidiomycota* were abundant to some extent in groups 3 and 4, respectively. Other genera included *Alternaria, Aspergillus, Penicillium, Myrothecium, Podospora* and others (Fig. 2).

### Analysis of fungal community composition

Fungal community analysis using principle coordinate analysis (Supplementary Figs S3–S5) of the 17 samples based on weighted UniFrac distances, unweighted UniFrac distances and Bray-Curtis analysis showed the separation of the samples into different groups. Unweighted analysis separated most members of root groups into locations from which they were collected. This was supported by the significant level of separation among groups using ADONIS (P = 0.0010). However, ADNOIS analysis showed that neither weighted UniFrac analysis (P = 0.1460), nor Bray-Curtis analysis (P = 0.2670) separated members of the different groups based on locations from which they were collected.

## Discussion

Limited studies in the past addressed fungal infections and endophytes in the roots of acid limes (*C. aurantifolia*). *Lasiodiplodia* species, *Neoscytalidium dimidiatum* and *Fusarium* species were common on acid lime roots^12,21,29^. A study by Michel-Rosales and Valdés^19^ and Reddy, *et al*.^20^ revealed colonization of *C. aurantifolia* roots with several arbuscular mycorrhizae. Mycorrhizae is common in the roots of several citrus species and it contributes to enhancing tolerance of citrus plants to drought, salinity, high temperature and diseases^17,18,30–32^. A part from lime, a study on other Citrus species using MiSeq analysis revealed association of several fungal taxa with *Citrus unshiu* roots^33^. Our study is the first to use MiSeq for the characterization of fungal communities in acid lime roots and the first to provide a quantitative analysis of fungal communities in acid lime roots.

Findings from our study revealed that the most dominant phylum in the roots is *Ascomycota*, made up mainly of the *Sordariomycetes* class and the *Fusarium* genus. Ascomycota is a large phylum of fungi, and its high presence in acid lime roots is not unexpected. *Fusarium* has been reported previously from roots of several citrus species^21,34^. However, the high dominance of Fusarium in acid lime roots is surprising. The results showed that in several roots, 95% of the root fungi are mainly *Fusarium*. This not only true in one place, but even in places which are apart from each other by more than 300 Km. This could be related to the fact that *Fusarium* growth rate is usually higher compared to several fungal species^35,36^. In addition, *Fusarium* species are well known to produce several toxic compounds which limit growth of other fungal species^37,38^, thus giving *Fusarium* a chance to dominate. However, a future study might be required to understand the reasons behind the dominance of *Fusarium* in acid lime roots.

Other fungal genera were detected in the acid lime roots. However most of the detected fungi were saprophytic and they are possibly endophytes interacting with the root system of acid limes. These included *Penicillium, Aspergillus*, *Glomus*, *Dactylella, Arthrobotrys* and *Rhizophagus*. *Glomus* and *Rhizophagus* are large genera of arbuscular mycorrhizae, commonly found associated with several plant species including citrus^18,19,30,39^. They help in nutrient absorption and alleviating drought and salinity stresses. Arbuscular mycorrhizal fungi (AMF) are common in plant roots and they enhance and modulate plant growth and tissue nutrient content which lead to an increase in their survival rates under various stressful conditions^2,40–42^. They can also provide bioprotection against biotic and abiotic stresses such as pathogens, pests, drought, salinity^42–44^.

Findings from the study revealed the presence of *Dactylella* and *Arthrobotrys*. These are genera of fungi with many species attacking and killing nematodes^45–49^. *Penicillium* and *Aspergillus* are weak and saprophytic fungal genera. They have been reported to interfere with the growth of other fungal pathogens^13,14,50,51^.

Weighted UniFrac distances and Bray-Curtis analysis showed that none of the two analyses separated the 17 acid lime samples according to places from which they were collected. This indicates that the samples do not show differences in the level of fungal diversity (weighted UniFrac) or according to their phylogenetic relationships (Bray-Curtis). On the other hand, unweighted UniFrac distances separated most samples according to the locations from which they were collected. Separation of samples based on Unweighted UniFrac distances gives an indication that fungal communities in each place are different to some extent from fungal communities from other places. The effects of location on the type of fungal taxa has been observed in other studies with other crops^52,53^.

MiSeq was able to recover some of the fungal genera which are usually uncultureable on synthetic media. These included *Glomus* and *Zopfiella*^30,32,54^. MiSeq is a powerful tool in the analysis of fungal communities, and it has been used previously for the detection and estimation of fungal diversity in several plant roots, leaves and stems^52,54–56^.

This study is the first high throughput sequencing approach that provides analysis of fungal diversity in acid lime roots. It shows the dominance of *Fusarium* in acid lime roots and the presence of several genera containing beneficial fungi. Future studies should investigate the reasons behind the dominance of *Fusarium*. In addition, the frequency of recovery of some fungal taxa was low. However, the roles played by such taxa cannot be ignored and future studies are required on the roles played by the different taxa in the root system of acid limes.

## Methods

### Collection of samples

**Figure 3:**
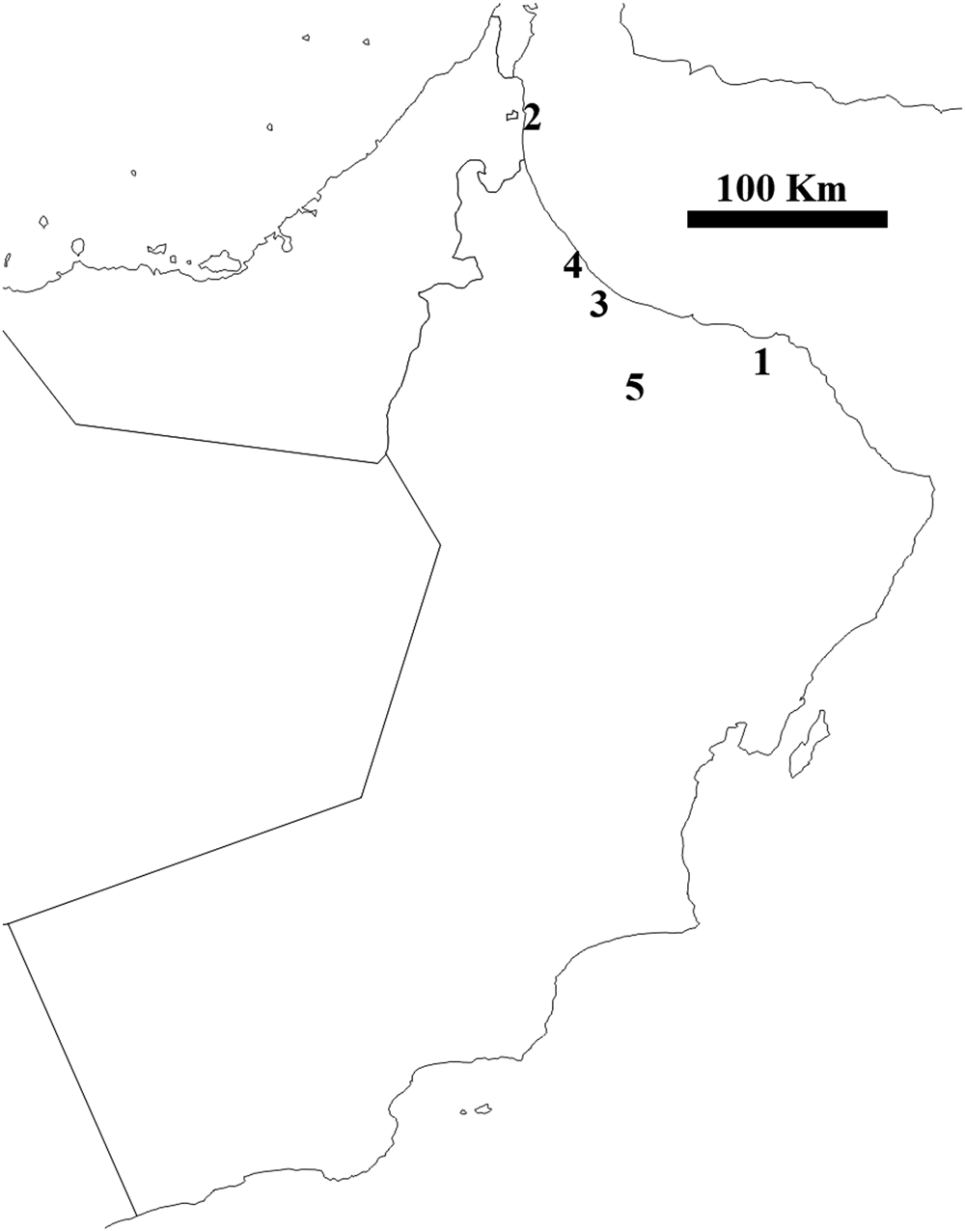
A map of Oman indicating the 5 locations from which root samples of acid limes were collected.

The study addressed fungal communities associated with roots of acid lime trees grown in five locations in Oman, 20–300 Km apart (Fig. 3). Two to four random acid lime-growing farms were selected from each location (total = 17). Root samples (3–10 mm thickness x 40–50 mm length) were collected from the top 30 cm of soil level of 6–8-year-old acid lime trees. Only apparently healthy root segments with no visual disease symptoms were collected. Roots were collected from three places around each acid lime tree, within 50–100 cm of the trunk. Root samples from each tree were placed in a separate sterile plastic bag. Samples were then transferred to Sultan Qaboos University and preserved at −80 °C. Roots were processed within five days of collection under sterile conditions.

### DNA extraction

Firstly, root samples were washed thoroughly using sterile distilled water to get rid of excess soil particles. Then, root samples were cut into 5–10 mm segments and ground using liquid nitrogen. DNA extraction from the ground root samples was done following the CTAB method of Doyle and Doyle^57^. The ground root samples were mixed with CTAB extraction buffer and the mixture was incubated at 65 °C for 10 min and immediately placed on ice for 10 min. An equal amount of phenol: chloroform: isoamyl alcohol (25:24:1) was added to each tube and mixed gently. The tubes were centrifuged and the previous step was repeated. Then 0.6 volume of isopropanol and 0.3 M NaAc (pH 5.2) was added to the supernatant and placed at −20 °C overnight. Samples were then centrifuged and the supernatant were discarded. The DNA pellets were washed twice with 70% ethanol followed by air-drying at room temperature. The DNA pellet was dissolved in 50 µl sterilized distilled water and stored at −80 °C until used. DNA concentration and quality were measured using a NanoDrop 2000 spectrophotometer (Thermo Scientific, USA).

### Illumina MiSeq analysis

The 17 DNA samples were subjected to Illumina MiSeq analysis (MISeq ITS1 assay; 10 K). Firstly, samples were amplified using the forward primer constructed from Illumina i5 sequencing primer (TCGTCGGCAGCGTCAGATGTGTATAAGAGACAG) and the ITS1F primer (CTTGGTCATTTAGAGGAAGTAA)^58^ and the reverse primer constructed from Illumina i7 sequencing primer (GTCTCGTGGGCTCGGAGATGTGTATAAGAGACAG) and ITS2aR primer (GCTGCGTTCTTCATCGATGC)^52,59^. The PCR reaction mixture consisted of DNA, 1 µl of each 5 µM primer and Qiagen HotStar Taq master mix (Qiagen Inc, Valencia, California). The reaction conditions included denaturation at 95 °C for 5 min, followed by 25 cycles of denaturation at 94 °C for 30 sec, annealing at 54 °C for 40 sec, and extension at 72 °C for 1 min (final for 10 min). Then a second PCR was conducted using the Illumina Nextera PCR primers and the same conditions, except for doing using 10 cycles^52^.

The amplified products were pooled and the size selection of each pool was done in two rounds. This was followed by quantification using the Quibit 2.0 fluorometer (Life Technologies) followed by loading on an Illumina MiSeq (Illumina, Inc. San Diego, California)^60^.

### Bioinformatic Analysis

The coverage of the Illumina MiSeq was 10000–20000 reads. Samples were assembled into OTU clusters at 97% identity using the USEARCH^61^ algorithm and then aligned using the UPARSE algorithm^62^ against a database of high quality ITS sequences from GenBank. Then a phylogenetic tree was constructed in Newick format from a multiple sequence alignment of the OTUs in MUSCLE^63,64^ and generated in FastTree^65^. Redundant sequences of the same species were removed. Fungi were classified at the appropriate taxonomic levels using trimmed taxa. The percentage of sequences assigned to each phylogenetic level were individually analyzed for each pooled sample providing relative abundance information within and among the individual samples.

The generated data were analyzed using the R software by generating a rarefaction curve plot of the number of OTUs versus the number of sequences^66^. Richness and Shannon Diversity indices were determined as explained by Kazeeroni and Al-Sadi^67^. Fungal community structure was analyzed using weighted UniFrac distances, unweighted UniFrac distances and Bray-Curtis analysis. Then differences in fungal diversity among groups from different locations were analyzed using ‘Permutational Multivariate Analysis of Variance Using Distance Matrices’ function ADONIS^68–70^.

## Electronic supplementary material

Supplementary Dataset 1

## Data Availability

All data underlying this publication are available in the manuscript.
