# Supplementary material for: Illumina-MiSeq analysis of fungi in acid lime roots reveals dominance of Fusarium and variation in fungal taxa
Source: Sci Rep. 2018 Nov 26;8:17388. doi: 10.1038/s41598-018-35404-5 (PMC6255777; doi:10.1038/s41598-018-35404-5)
Supplement: Supplementary file 1 — Supplementary Dataset 1 [file 41598_2018_35404_MOESM1_ESM.pdf]

Illumina-MiSeq analysis of fungi in acid lime roots reveals dominance of *Fusarium* and variation in fungal taxa

Abdullah M. Al-Sadi and Elham A. Kazerooni

Department of Crop Sciences, College of Agricultural and Marine Sciences, Sultan Qaboos University, Oman, PO Box 34, Alkhoud 123, Oman

Supplementary figures

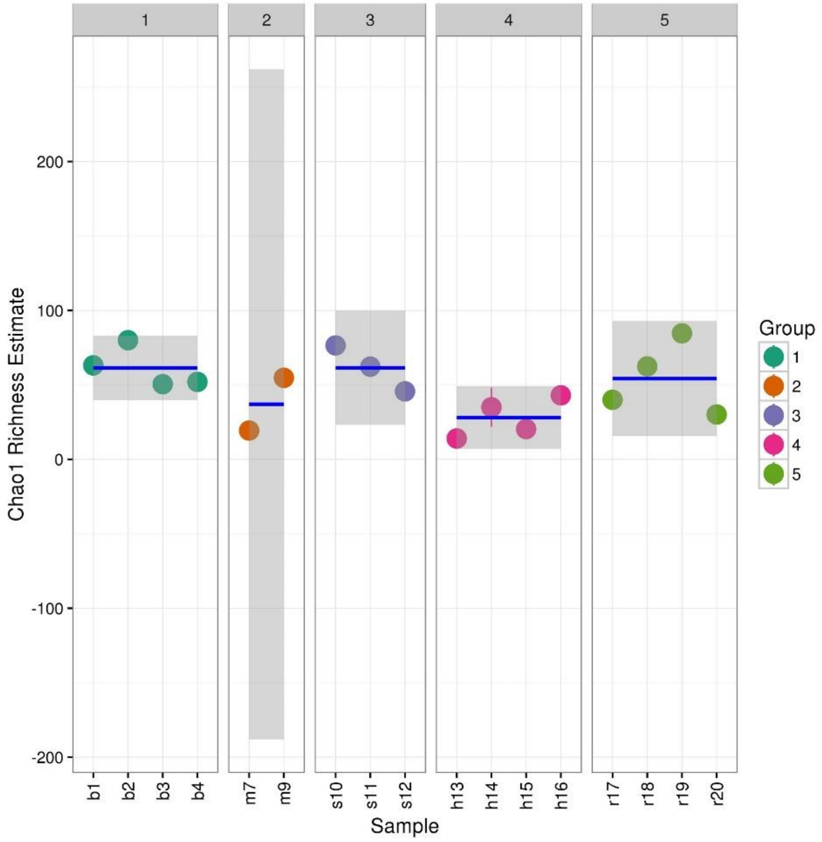

Supplementary Fig. S1 Chao1 richness of the five groups of acid lime roots.

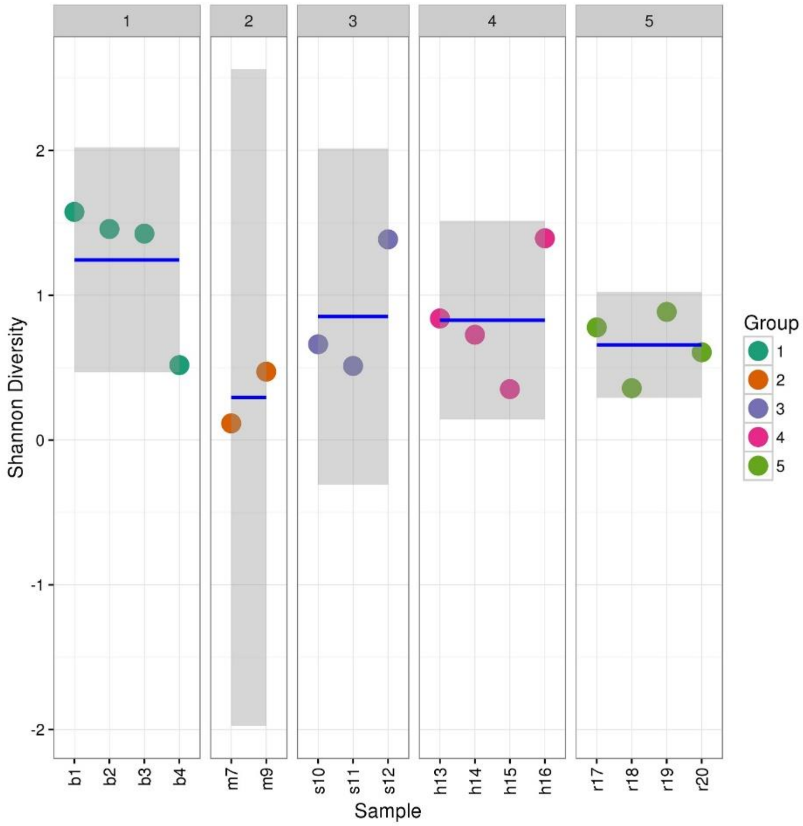

Supplementary Fig. S2 Shannon diversity of the five groups of acid lime roots.

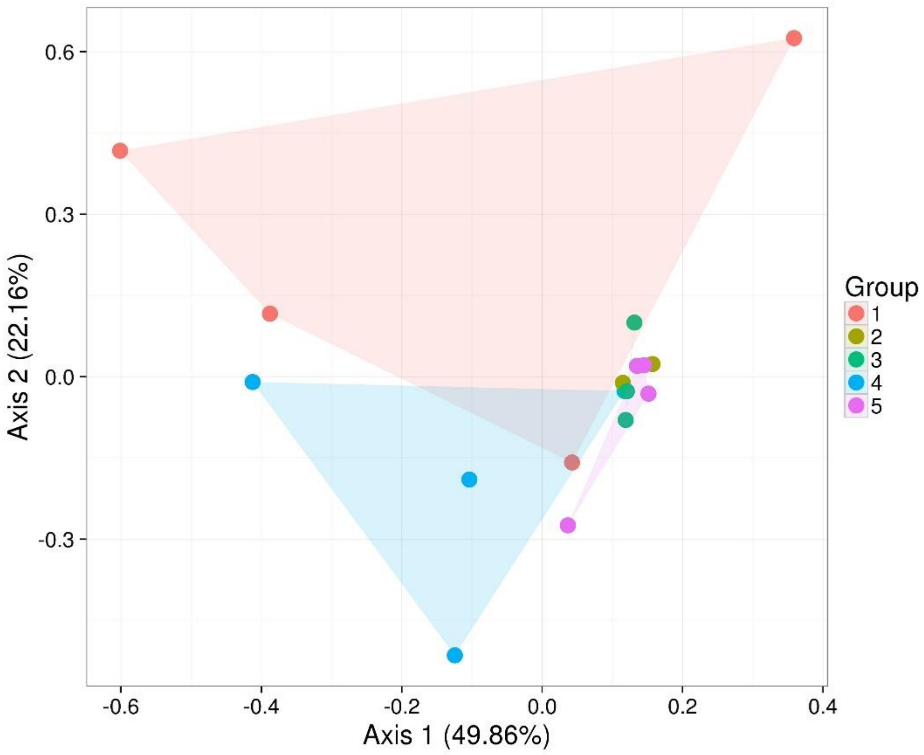

Supplementary Fig. S3 Principle component analysis of the relative abundances of the dominant fungal genera in the five groups of lime roots (17 samples) based on weighted UniFrac distances.

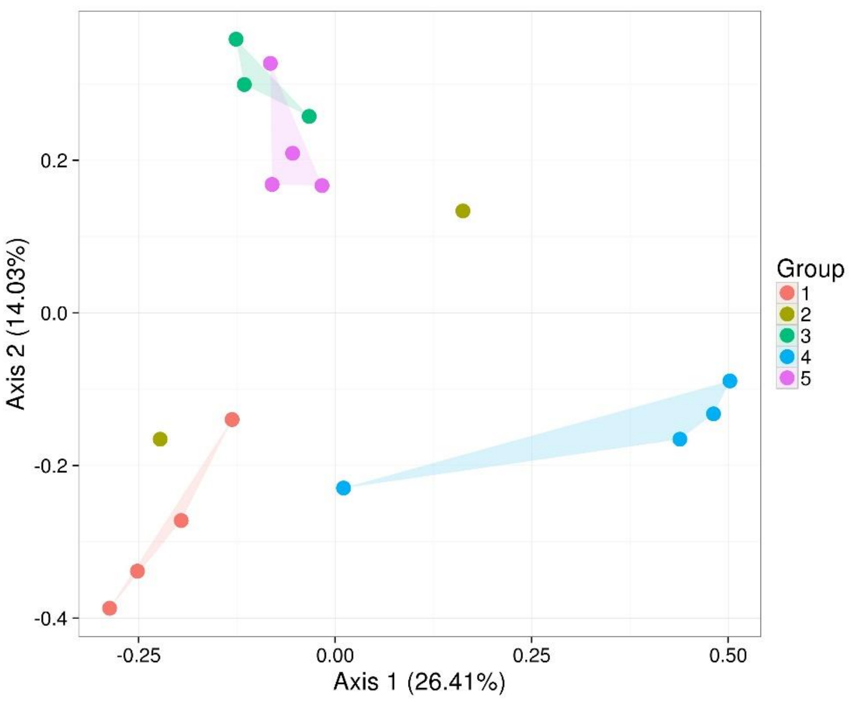

Supplementary Fig. S4 Principle component analysis of the relative abundances of the dominant fungal genera in the five groups of lime roots (17 samples) based on unweighted UniFrac distances.

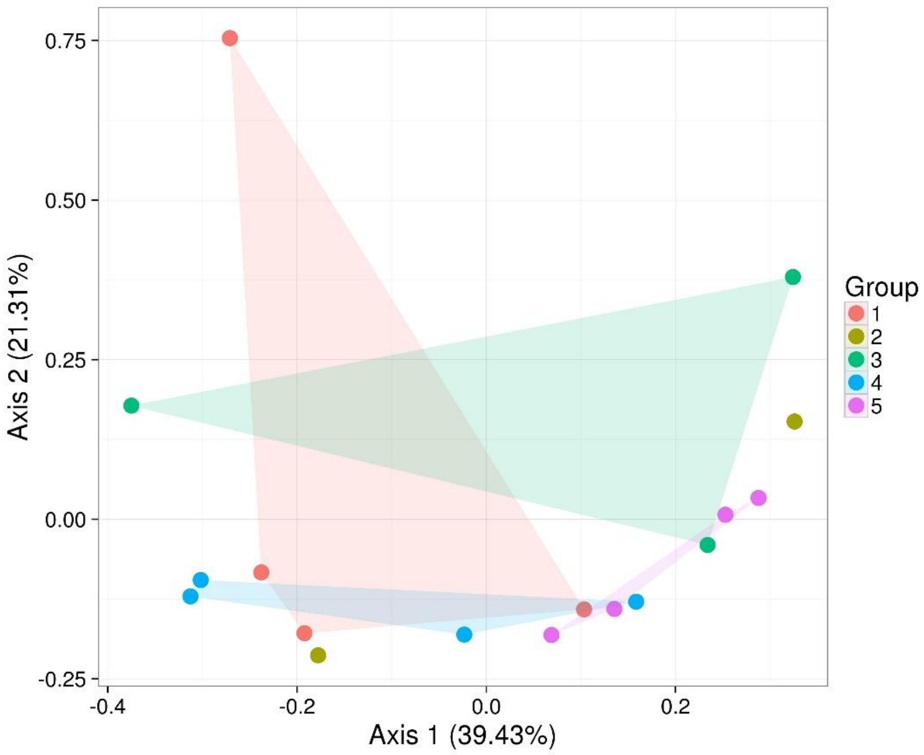

Supplementary Fig. S5 Principle component analysis of the relative abundances of the dominant fungal genera in the five groups of lime roots (17 samples) based on Bray-Curtis analysis.
